# Metabolite signature of human malignant thyroid tissue: A systematic review and meta‐analysis

**DOI:** 10.1002/cam4.7184

**Published:** 2024-04-22

**Authors:** S. Adeleh Razavi, Babak Khorsand, Pouya Salehipour, Mehdi Hedayati

**Affiliations:** ^1^ Cellular and Molecular Endocrine Research Center, Research Institute for Endocrine Sciences Shahid Beheshti University of Medical Sciences Tehran Iran; ^2^ Department of Neurology University of California Irvine California USA; ^3^ Department of Computer Engineering, Faculty of Engineering Ferdowsi University of Mashhad Mashhad Iran; ^4^ Department of Medical Genetics, School of Medicine Tehran University of Medical Sciences Tehran Iran

**Keywords:** meta‐analysis, metabolomics, NMR, thyroid carcinoma, thyroid lesions

## Abstract

**Background:**

Thyroid cancer (TC) is the predominant malignancy within the endocrine system. However, the standard method for TC diagnosis lacks the capability to identify the pathological condition of all thyroid lesions. The metabolomics approach has the potential to manage this problem by identifying differential metabolites.

**Aims:**

This study conducted a systematic review and meta‐analysis of the NMR‐based metabolomics studies in order to identify significant altered metabolites associated with TC.

**Methods:**

A systematic search of published literature in any language in three databases including Embase, PubMed, and Scopus was conducted. Out of 353 primary articles, 12 studies met the criteria for inclusion in the systematic review. Among these, five reports belonging to three articles were eligible for meta‐analysis. The correlation coefficient of the orthogonal partial least squares discriminant analysis, a popular model in the multivariate statistical analysis of metabolomic data, was chosen for meta‐analysis. The altered metabolites were chosen based on the fact that they had been found in at least three studies.

**Results:**

In total, 49 compounds were identified, 40 of which were metabolites. The increased metabolites in thyroid lesions compared normal samples included lactate, taurine, alanine, glutamic acid, glutamine, leucine, lysine, phenylalanine, serine, tyrosine, valine, choline, glycine, and isoleucine. Lipids were the decreased compounds in thyroid lesions. Lactate and alanine were increased in malignant versus benign thyroid lesions, while, myo‐inositol, scyllo‐inositol, citrate, choline, and phosphocholine were found to be decreased. The meta‐analysis yielded significant results for three metabolites of lactate, alanine, and citrate in malignant versus benign specimens.

**Discussion:**

In this study, we provided a concise summary of 12 included metabolomic studies, making it easier for future researchers to compare their results with the prior findings.

**Conclusion:**

It appears that the field of TC metabolomics will experience notable advancement, leading to the discovery of trustworthy diagnostic and prognostic biomarkers.

## Graphical abstract

We present a systematic review and meta‐analysis of the current literature on the metabolite signature of human thyroid cancer. We have conducted a systematic search of the Embase, PubMed, and Scopus databases to identify relevant studies. We have then performed a comprehensive qualitative review and a brief meta‐analysis to identify metabolites that were consistently altered in thyroid cancer. The study indicates that several metabolites belonging to the various metabolic pathways altered in thyroid lesions, particularly thyroid cancer.

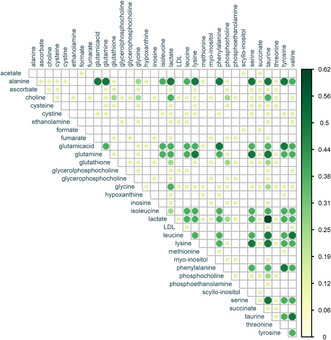

## 1 INTRODUCTION

The common definition for metabolomics is the large‐scale analysis of small molecules, called metabolites, with a molecular weight of <1500 daltons (Da). The metabolomics approach can be used in various biological samples such as biological fluids (whole blood, serum, plasma, urine, saliva, sweat, milk, semen, etc.), tissues, cells, plants, and foods.^1^, ^2^ What makes metabolomics important is that it is the final downstream omics that brings researchers very close to the molecular phenotype understanding of an organism. In this regard, it can represent the metabolic differences between health and disease and can be applied in precision medicine.^1^ Examining the human metabolome can open the new windows for identification of metabolites with promising applications in diagnosis, prognosis, and treatment of different diseases. In fact, metabolic profiles have the capacity to function as potential biomarkers for the early diagnosis, progression, and outcomes of different diseases, encompassing cancer. On the other hand, the metabolites identification can lead to targeted treatments that take advantage of metabolic pathways.

Due to the complexity of the metabolome, robust analytical techniques are required for quantitative analysis. The most common techniques used in metabolomics are nuclear magnetic resonance (NMR), liquid chromatography‐mass spectrometry (LC‐MS), and gas chromatography‐mass spectrometry (GC‐MS), which allow identification and quantification of metabolites.^3^, ^4^

NMR utilizes the magnetic characteristics of specific atomic nuclei, such as ^1^H and ^13^C found in molecules. By subjecting these nuclei to a powerful magnetic field and exposing them to radio frequency pulses, they absorb and subsequently emit electromagnetic radiation at distinct frequencies. As a result, a distinct identifier is generated for each metabolite present in a sample. NMR spectroscopy is an essential instrument for studying metabolomics, providing significant benefits in terms of non‐invasive, quantitative, and consistent examination of metabolites within intricate biological samples. Consequently, it plays a vital role in enhancing the comprehension of metabolic pathways and their impact on diverse physiological and pathological states. Hence NMR‐based metabolomics is beneficial and practical for extensive research in the field of cancer.^5^, ^6^

Among all human cancers that have been studied by NMR‐based metabolomics approach, the contribution of thyroid cancer studies is restricted. Meanwhile, thyroid cancer is the most common endocrine malignancy and its prevalence is increasing.^7^ Diagnosis of thyroid cancer is performed by the cytological examination of fine needle aspiration biopsy (FNAB) which is the most common and efficient method for preoperative diagnosis of the nature of thyroid nodules.

Thyroid FNAB examination, with 90% accuracy, is able to discriminate 70% of specimens as benign or malignant nodules. However, the important limitation of the method is that it is not able to differentiate follicular adenomas from carcinomas in 10%–30% of cases. These cases, which have an indeterminate cytology, undergo diagnostic surgery. But the rate of malignancy in this group is about 14%–20%, which shows that 80% of patients undergo unnecessary surgery.^8^, ^9^

The American Thyroid Association (ATA) has suggested that researchers look for biomarkers that have the power of differential diagnosis.^10^ Metabolomics approach has a high potency to identify biomarkers in the context of screening and diagnosis of cancer. So, the problem of thyroid FNABs with indeterminate cytology may be managed by the metabolomics capabilities. Distinguishing metabolic alterations between cancerous and non‐cancerous thyroid lesions has the potential to facilitate the creation of more precise and delicate diagnostic techniques. This could potentially decrease the occurrence of misdiagnoses and avoid unnecessary surgical procedures. Additionally, it would decrease the necessity for invasive methods and mitigate the accompanying risks.

The main objective of the current study was to systematically review studies that aimed to identify altered metabolites for discriminating between thyroid lesions using an NMR‐based metabolomics approach. In this regard, we performed a systematic review and meta‐analysis to answer the following two focused questions: (1) According to the NMR‐based metabolomics, which metabolites are significantly higher/lower in human thyroid lesions versus normal tissues, as well as human thyroid cancer versus benign tissues? (2) Do these metabolites possess the ability to differentiate malignancy from benignity?

## 2 METHODS

### 2.1 Data sources and search strategy

This systematic review and meta‐analysis has been registered at the International Prospective Register of Systematic Reviews (PROSPERO) with the registration ID number of CRD42022370330. PROSPERO functions as a thorough and varied database committed to the advance registration of protocols for systematic reviews. The main aim of PROSPERO is to promote transparency, reduce bias, and improve the caliber of systematic reviews in diverse areas of research, particularly in healthcare, and social interventions.

We conducted a systematic search of published literature in any language in three different databases (Embase, PubMed, and Scopus) from inception to November 1, 2022, by use of the following approach. We used the Preferred Reporting Items for Systematic Reviews and Meta‐Analyses (PRISMA) method for the systematic reviews. The reference lists of the selected articles were also hand‐searched.

#### 2.1.1 Keywords and their connections

The following keywords with their respective connections used for references identification. Instructions on how to search each of the databases are provided in the Data S1. No exclusion criteria were applied during this step (“metabolomics” OR “metabonomics” OR “metabolome” OR “metabolic profile” OR “metabolomic profile” OR “metabolites profile”) AND (“thyroid neoplasm” OR “thyroid carcinoma” OR “thyroid cancer” OR “thyroid adenoma” OR “thyroid nodule” OR “thyroid tumor” OR “nodular goiter” OR “multinodular goiter” OR “multi‐nodular goiter” OR “MNG”).

### 2.2 Study selection and eligibility criteria

Following references identification, a meticulous screening process was initiated to ensure the inclusion of relevant studies in the analysis. Initially, all identified studies were imported into the Endnote software for systematic management. Subsequently, any duplicate publications were removed. The screening phase then commenced, where the titles, abstracts, and keywords of the remaining studies underwent thorough evaluation by two reviewers (S. Adeleh Razavi and Mehdi Hedayati). This rigorous dual‐review process aimed to meticulously assess each study's alignment with the research focus on thyroid cancer and metabolomics and relevance to the study objectives. Study exclusions encompassed irrelevant studies, conference abstracts, non‐original papers such as reviews, editorials, and book chapters, as well as studies focusing on animal, plant, or cellular models. Furthermore, non‐English studies were also omitted from the analysis.

**FIGURE 1:**
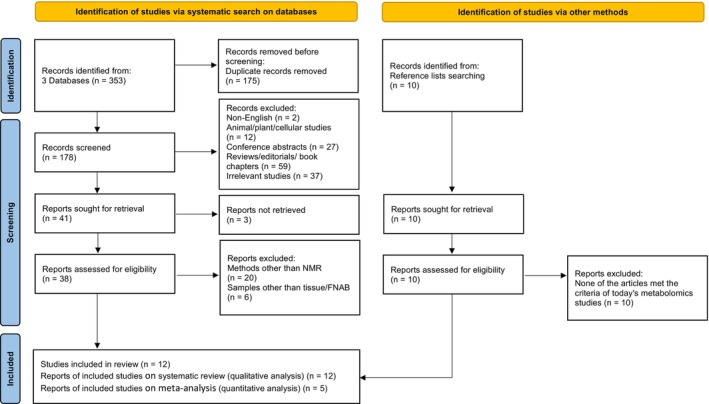
Flow chart of study selection for the systematic review and meta‐analysis.

Given the diverse range of specimen types, including serum, plasma, tissue, among others, and the various analytical platforms prevalent in metabolomics research (such as GC–MS, LC–MS, NMR), interpreting and synthesizing research findings can pose significant challenges. To ensure homogeneity in the data analyzed, stringent eligibility criteria were established for study inclusion. Specifically, the scope of the review was limited to encompass tissue and fine‐needle aspiration biopsy (FNAB) specimens analyzed using the NMR analytical platform. Throughout the study selection process, any discrepancies were meticulously addressed through scientific deliberation among the research team. The flow chart of study selection is presented in Figure 1.

### 2.3 Quality assessment and data extraction

The quality of the included studies was determined using the case–control version of “The Newcastle‐Ottawa Scale (NOS)” for assessing the quality of non‐randomized studies in meta‐analyses (https://www.ohri.ca/programs/clinical_epidemiology/oxford.asp). The NOS evaluates the quality of the study based on the three main criteria of selection, comparability, and exposure. Selection (with four items) assesses how well the selection of cases and controls in individual studies is defined and whether they are representative of the target population. Comparability (with one item) assesses the comparability of cases and controls on important factors or characteristics. Exposure (with three items) assesses how the exposure was determined for the case and control groups. A study can be awarded a maximum of one star for each item within the selection and exposure categories. A maximum of two stars can be given for comparability. The exposure item was not applicable in this study. Therefore, only selection and comparability were evaluated. Accordingly, the highest possible score was 6 stars (Table 1).

**TABLE 1:** The Newcastle‐Ottawa quality assessment for the included studies.

Reference	Selection (up to four stars)				Comparability (up to two stars)	Exposure			Quality score (max = 6)
	Is the case definition adequate?	Representativeness of the cases	Selection of controls	Definition of controls	Comparability of cases and controls on the basis of the design or analysis	Ascertainment of exposure	Same method of ascertainment for cases and controls	Non‐response rate	
Jordan, 2011^11^	*	*	—	*	**	NR	NR	NR	*****
Miccoli, 2012^12^	*	*	—	*	**	NR	NR	NR	*****
Torregrossa, 2012^13^	*	*	—	*	**	NR	NR	NR	*****
Deja, 2013^14^	*	*	—	*	**	NR	NR	NR	*****
Tian, 2015^15^	*	*	—	*	**	NR	NR	NR	*****
Lu, 2016^16^	*	*	—	*	*	NR	NR	NR	****
Ryoo, 2016^17^	*	*	—	*	*	NR	NR	NR	****
Li, 2018^18^	*	*	—	*	*	NR	NR	NR	****
Rezig, 2018^19^	*	*	—	*	**	NR	NR	NR	*****
Seo, 2018^20^	*	*	—	—	—	NR	NR	NR	**
Metere, 2020^21^	*	*	—	—	*	NR	NR	NR	***
Skorupa, 2021^22^	*	*	—	*	**	NR	NR	NR	*****

Abbreviation: NR, not relevant to this study.

For the eligible studies, data were extracted using data extraction form that included the following items. Authors and year of publication, country where the research was conducted, number of participants and their age, type of sample (e.g., intact tissue, tissue extract, FNAB, and etc.) and how to collect it, the method of examining the pathological condition (e.g., pre or postoperative histopathological examination, and etc.), the number and type of studied samples based on the pathology report (Table 2). Methodology information (e.g., approach, type of NMR instrument, the field strength of the NMR magnet, and etc.) was recorded in a separate form (Table 3).

**TABLE 2:** Characteristics of selected studies for systematic review and meta‐analysis.

Reference	Country	No. of patients (Female/male)	Age^a^	Type of sample	Sample collection	Pathologic review	No. and cyto/histopathology of specimens
Jordan, 2011^11^	USA	ND	ND	Intact tissue & FNAB	Tissue: surgical specimen FNAB: ex‐vivo aspiration Storage: snap‐freezing then storing at −80°C	Postoperative histopathological examination	Pairs of FNAB and tissue: 4 pairs of PTC 4 pairs of FA 5 pairs of normal (uninvolved tissue by FA or PTC)
Miccoli, 2012^12^	Italy	72 (47/25)	42.8 ± 17.9 (9–88)	Intact tissue & FNAB	Tissue: surgical specimen FNAB: ex‐vivo aspiration Storage: snap‐freezing in liquid nitrogen then storing at −80°C	Postoperative histopathological examination	28 PTC 40 indeterminate 4 benign 28 normal (controlateral healthy thyroid tissue) 12 FNAB
Torregrossa, 2012^13^	Italy	72 (47/25)	42.8 ± 17.9 (9–88)	Intact tissue	Tissue: surgical specimen Storage: snap‐freezing in liquid nitrogen then storing at −80°C	Postoperative histopathological examination	27 PTC 1 ATC 10 FTC 30 FA 4 NG 28 Normal (controlateral healthy thyroid tissue)
Deja, 2013^14^	Poland	31 (4/27)	53.6 ± 15.8	Tissue extract	Tissue: surgical specimen Storage: snap‐freezing in liquid nitrogen then storing at −80°C	Postoperative histopathological examination	16 non‐neoplastic nodules 14 FA 15 thyroid carcinoma 19 normal (controlateral healthy thyroid tissue)
Tian, 2015^15^	China	53 (44/9)	21–75	Intact frozen tissue	Tissue: surgical specimen Storage: snap‐freezing in liquid nitrogen then storing at −80°C	Postoperative histopathological examination	22 NG 3 FA 28 PTC 46 normal (healthy adjacent thyroid tissue)
		50 (43/7)	21–71	Tissue extract			22 NG 3 FA 25 PTC 46 normal (healthy adjacent thyroid tissue)
Lu, 2016^16^	China	42 (36/6)	45.1 ± 8.5	Intact frozen tissue	Tissue: surgical specimen Storage: snap‐freezing in liquid nitrogen then storing at −80°C	Postoperative histopathological examination	16 micro PTC 11 normal (healthy nearby thyroid tissue)
Ryoo, 2016^17^	Korea	100 (77/23)	52.9 ± 10.8 (21–77)	FNAB	FNAB: in‐vivo aspiration Storage: maintenance in dry‐ice box then storing at liquid nitrogen tank	Preoperative cytopathological examination	69 benign (FA) 35 PTC
Li, 2018^18^	China	16 (12/4)	19–59	Intact tissue	Tissue: surgical specimen Storage: snap‐freezing in liquid nitrogen then storing at −80°C	Postoperative histopathological examination	16 PTC 16 normal (matched thyroid tissue)
Rezig, 2018^19^	Italy	96 (72/24)	48 ± 14 (20–79)	FNAB	FNAB: ex‐vivo aspiration Storage: snap‐freezing in liquid nitrogen then storing at −80°C	Postoperative cytopathological examination	46 benign (6 Goiter, 39 FA, 1 lymphocytic thyroiditis) 52 malignant (50 PTC, 1 FTC, 1 MTC)
Seo, 2018^20^	Korea	52 (40/12)	15–74	Tissue extract	Tissue: surgical specimen Storage: snap‐freezing in liquid nitrogen then storing at −70°C	ND	32 PTC with central LN metastasis 20 PTC without LN metastasis 19 PTC with lateral LN metastasis 33 PTC without lateral LN metastasis
Metere, 2020^21^	Italy	14 (12/2)	26–64	Tissue extract	Tissue: surgical specimen Storage: quickly frozen (within 30 min) then storing at −80°C	Postoperative histopathological examination	11 thyroid cancer 10 normal (5 matched normal thyroid tissues and 5 benign tissues)
Skorupa, 2021^22^	Poland	141 (113/28)	53.3 ± 15.8	Intact frozen tissue	Tissue: surgical specimen Storage: snap‐freezing on dry ice then storing at −80°C	Postoperative histopathological examination Post‐NMR histopathological examination	38 PTC 32 benign lesions 112 non‐tumoral tissue (80 colloid goiter and 32 chronic thyroiditis)

Abbreviations: ATC, anaplastic thyroid carcinoma; FA, follicular adenoma; FNAB, fine needle aspiration biopsy; FTC, follicular thyroid carcinoma; LN, lymph node; MTC, medullary thyroid carcinoma; ND, not disclosed; NG, nodular goiter; PTC, papillary thyroid carcinoma.

^a^ Mean ± SD and/or range, years.

**TABLE 3:** Methodological information of selected studies for systematic review and meta‐analysis.

Reference	Type of study	Approach	Platform (instrument)	Pulse sequence method (echo time)	Metabolite assignment	Multivariate analysis	Univariate analysis	Statistical software/pathway analysis tool
Jordan, 2011	Case/Control	Profiling	HRMAS ^1^H NMR (Bruker Avance 600 MHz)	CPMG (100 ms)	ND	PCA	ANOVA Linear regression Canonical analysis Paired *t*‐test	JMP
Miccoli, 2012	Case/Control	Profiling	HRMAS ^1^H NMR (Bruker Avance 400 MHz)	CPMG (80 ms)	^1^H−^1^H TOCSY iNMR Literature	PCA OPLS‐DA	ROC curve	SIMCA‐P 12.0
Torregrossa, 2012	Case/Control	Profiling	HRMAS ^1^H NMR (Bruker Avance 400 MHz)	CPMG (80 ms)	^1^H−^1^H TOCSY ^1^H−^13^C HSQC iNMR In‐house databases Literature	PCA OPLS OPLS‐DA	ROC curve	SIMCA‐P 12.0 MATLAB 7.4
Deja, 2013	Case/Control	Profiling	^1^H NMR (Bruker Avance II 600 MHz)	CPMG (400 μs)	HMDB^a^ BMRB^b^ ^1^H–^1^H COSY ^1^H–^1^H TOCSY ^1^H–^13^C HSQC In‐house databases	PCA OPLS‐DA	Kruskal–Wallis one‐way ANOVA Student *t*‐test Mann–Whitney‐Wilcoxon test	SIMCA‐P+ 13.0 STATISTICA 10 MSEA^c^
Tian, 2015	Case/Control	Profiling	HRMAS ^1^H NMR (Bruker Avance III 600 MHz)	CPMG (ND)	^1^H–^1^H COSY ^1^H–^1^H TOCSY ^1^H JRES ^1^H–^13^C HSQC ^1^H−^13^C HMBC	PCA OPLS‐DA CV‐ANOVA	ROC curve	SIMCA‐P+ 12.0 MATLAB SPSS 18.0
Lu, 2016	Case/Control	Profiling	HRMAS ^1^H NMR (Bruker Avance III 800 MHz)	CPMG (ND)	Chenomx NMR Suite 8.1 ^1^H ‐^1^H COSY ^1^H ‐^1^H TOCSY ^1^H‐^13^C HSQC	PCA PLS‐DA	**–**	R
Ryoo, 2016	Case/Control	Profiling	^1^H NMR (Bruker Avance III 700 MHz)	CPMG (ND)	Chenomx In‐house Perl program	OPLS‐DA	Unpaired student *t*‐test Wilcoxon rank‐sum test ROC curve	SIMCA‐P 11.0 R ROC curve explorer & tester
Li, 2018	Case/Control	Profiling	^1^H NMR (Bruker Avance III 600 MHz)	CPMG (ND)	ND	PCA PLS‐DA OPLS‐DA	**–**	SIMCA‐P+ 11.0 KEGG^d^ HMDB SMPDB^e^ MetaboAnalyst 3.0
Rezig, 2018	Case/Control	Profiling	HRMAS ^1^H NMR (Bruker Avance III 400 MHz)	CPMG (60 ms)	^1^H–^1^H TOCSY ^1^H–^13^C HSQC In‐house and online databases Literature	PCA OPLS‐DA	ANOVA ROC curve	SIMCA‐P+ 14.0
Seo, 2018	Case/Control	Profiling	^1^H NMR (Bruker Avance 700 MHz)	CPMG (2 ms)	Chenomx NMR Suite 7.7	OPLS‐DA	Chi‐square analysis Student *t*‐test Mann‐Whitney U test	MATLAB R2012a SIMCA‐P 13.0 Excel R Package 3.3.3
Metere, 2020	Case/Control	Profiling	^1^H NMR (Bruker Avance 400 MHz)	60º flip angle pulse	XWIN‐NMR HMDB In‐house previous studies	PLS‐DA	ANOVA FDR	MetaboAnalyst 4.0 SPSS 23
Skorupa, 2021	Case/Control	Profiling	HRMAS ^1^H NMR (Bruker Avance III 400 MHz)	CPMG (60 ms)	^1^H JRES ^1^H–^1^H TOCSY ^1^H–^13^C HSQC Literature	PCA OPLS‐DA CV‐ANOVA SUS plot	Kruskal–Wallis test	SIMCA‐P 15.0 STATISTICA 12.5 MetaboAnalyst 4.0

*Note*: For other abbreviations, refer to the text.

Abbreviation: ND, not disclosed.

^a^ Human Metabolome Database (http://hmdb.ca/).

^b^ Biological Magnetic Resonance Bank (http://bmrb.io/).

^c^ Metabolite Set Enrichment Analysis (http://msea.ca/).

^d^ Kyoto Encyclopedia of Genes and Genomes (http://kegg.jp/).

^e^ Small Molecule Pathway Database (http://smpdb.ca/).

For each study, the number and type of identified metabolites, their increased or decreased level, and all presented results from multivariate, and univariate analyses were recorded. Association between the level of a given metabolite with malignancy or benignity that reported through correlation coefficient, fold change, receiver operating characteristic curve, and etc. were recorded carefully and with all the details.

### 2.4 Data synthesis and meta‐analysis

First, a comprehensive qualitative review was conducted on all included studies (12 articles). The number and type of metabolites identified in each study were represented using frequency charts to introduce general trends. The main metabolites were then meta‐analyzed based on the reported correlation coefficients in the studied groups.

It is important to mention that, because of the lack of adequate information, only five reports pertaining to three studies were incorporated in the meta‐analysis. Despite concerted efforts to request supplementary data, we did not receive a response, thereby the breadth and depth of the meta‐analytical investigation was limited. This limitation impeded the acquisition of additional datasets for comprehensive analysis.

Heterogeneity between studies (using *Q*‐test and *I* ^2^ statistics) and studies effect (using Forest plots) were assessed by the Comprehensive Meta‐Analysis (CMA V3) and MedCalc 19.2 software. Heterogeneity was considered statistically significant at *p* ≤ 0.10. The significance level of studies effect was defined by a *p* < 0.05.

With the aim of identifying the influencing metabolites, a bipartite network was drawn for the metabolites that were recognized in the systematic review. A bipartite network comprised of two distinct categories of nodes, wherein connections solely transpired between nodes of dissimilar categories. Within this investigation, the nodes exemplified two dissimilar categories of metabolites and studies. This form of network visualization led to the comprehension of connections between metabolites and studies. Next, bipartite network projection that is extensively used for compacting data about bipartite networks was obtained.^23^, ^24^, ^25^ Network projection was employed to streamline bipartite networks by converting them into unipartite networks. This conversion aided in the more efficient analysis of the relationships within the data. The correlation between two metabolites was calculated based on the percentage of co‐reported frequency of the two metabolites in the articles.^26^, ^27^, ^28^

## 3 RESULTS

### 3.1 Literature search results

In the systematic search of three databases (Embase, PubMed, Scopus), 353 articles were found, of which 174 were duplicates. After removing duplicates and removing non‐English papers, reviews/editorials/book chapters, conference abstracts, animal/plant/cellular studies, and irrelevant studies, 42 articles remained that their full text were read. Of these, three articles were not retrieved, because they used imaging methods or capillary electrophoresis. Out of 39 articles, 19 articles used a platform other than NMR (GC–MS or LC–MS), and eight articles studied samples other than tissue/FNAB (serum, plasma, etc.). Finally, 12 articles^11^, ^12^, ^13^, ^14^, ^15^, ^16^, ^17^, ^18^, ^19^, ^20^, ^21^, ^22^ were included in the qualitative study. Out of 12 articles and due to the limitations of the reported results, five reports related to three articles were selected for the quantitative study (Figure 1).

It should be noted that the references of 12 included studies were hand‐searched and 10 articles^29^, ^30^, ^31^, ^32^, ^33^, ^34^, ^35^, ^36^, ^37^, ^38^ apparently related to the topic were assessed for eligibility. After reading the full text, it was found that most of these articles are dated prior to 2000, and none of them possess the components that are present in today's untargeted metabolomics studies. For example, most of them investigated a specific region of the NMR spectrum (e.g., a ratio of the intensities of resonances at 0.9 ppm and 1.7 ppm)^29^, ^31^ and were not among metabolites profiling studies. There were no common multivariate analyses (e.g., principal component analysis, or orthogonal partial least squares discriminant analysis [OPLS‐DA]) in them either. Therefore, none of these 10 articles were recognized as eligible.

### 3.2 Eligible studies characteristics

The studies included in the current study were published between 2011 and 2021 and belonged to the countries of the USA (one article), Italy (four articles), Poland (two articles), China (three articles), and Korea (two articles). The highest number of study participants were 141 patients^22^ and the lowest number were 14 patients.^21^ Among the participants in the included studies, the minimum age was 9 years and the maximum age was 88 years.^12^, ^13^ The studies used different types of samples including FNAB, tissue extract and intact tissue. The number and cyto/histopathology of evaluated specimens are presented in Table 2. Except for the Seo et al. study^20^ that compared papillary thyroid carcinoma (PTC) patients with or without lymph nodes metastasis, other studies compared metabolite profiles between the thyroid lesions group and the normal group, as well as the malignant and benign groups. All studies were case–control studies and chose the metabolomic profiling approach. Seven studies used high‐resolution magic angle spinning ^1^H NMR (HRMAS ^1^H NMR) and five studies used ^1^H NMR. Except for Metere et al.'s study,^21^ the used pulse program of all studies was Carr‐Purcell‐Meiboom‐Gill pulse sequence. Other methodological information, such as metabolite assignment, multivariate analysis, univariate analysis, statistical software, and pathway analysis tool is fully presented in Table 3.

The quality assurance of included studies was performed based on the NOS method. Out of 12 reviewed studies, seven articles scored 5 stars, three articles scored 4 stars, one article scored 3 stars, and one article scored 2 stars. These stars were interpreted as follows: 5 stars: good, 3–4 stars: fair, and 0–2 stars: poor. Therefore, in this study, seven articles with good quality, four articles with fair quality and one article with poor quality were included (Table 1).

### 3.3 Systematic review results

Jordan and colleagues did not suggest specific metabolites in their results. They simply demonstrated, through univariate statistical analysis, that NMR‐based metabolomic profiles were sensitive enough to differentiate between normal, PTC and follicular adenoma (FA) in tissue and FNAB samples.^11^

In another study, Seo et al. conducted a comparison between two groups of PTC patients, one with metastasis to lymph nodes and the other without, and found that there was no significant distinction in metabolic profile between the two groups. However, they proposed in this comparison, lactate was an important metabolite. In their subsequent study, Seo et al. compared PTC patients with lateral lymph nodes metastasis to those without, and similarly found no statistically remarkable difference in metabolic profiles between the groups. But they did identify lactate and myo‐inositol as important metabolites in patients with lateral lymph nodes metastasis.^20^

In their research, Metere et al. compared 11 samples of thyroid cancer and a control group comprising of both healthy and benign samples. This study showed that acetic acid, alanine, creatine, formic acid, glutathione, isoleucine, lactate, phenylalanine, and tyrosine were higher and citrate, myo‐inositol, and threonine were lower in the thyroid cancer group.^21^ Due to the inclusion of one of the thyroid lesions (benign tissues) in the control group, the results of this study are not incorporated in the subsequent sections.

The following sections present the results of the other studies that have clearly elucidated the differences in metabolites status among the studied groups (thyroid lesions vs. normal and malignant vs. benign thyroid tissues/FNABs).

#### 3.3.1 Altered metabolites in thyroid lesions versus normal

The number of individual compounds identified in nine studies^12^, ^13^, ^14^, ^15^, ^16^, ^17^, ^18^, ^19^, ^22^ was 49 that their common names, PubChem CID and Human Metabolome Database (HMDB) ID are listed in Table S1. Significant altered metabolites between thyroid lesions and normal specimens observed in included articles^12^, ^13^, ^14^, ^15^, ^16^, ^17^, ^18^, ^19^, ^22^ have been summarized using the vote‐counting charts. Each metabolite received one vote per article. The study conducted by Tian et al.^15^ used two different types of samples (intact tissue and tissue extract) and reported separate results for each type of sample. Accordingly, in the systematic review and meta‐analysis, these results have been evaluated as two separate reports under the title of Tian‐1 et al. (intact tissue) and Tian‐2 et al. (tissue extract).

**FIGURE 2:**
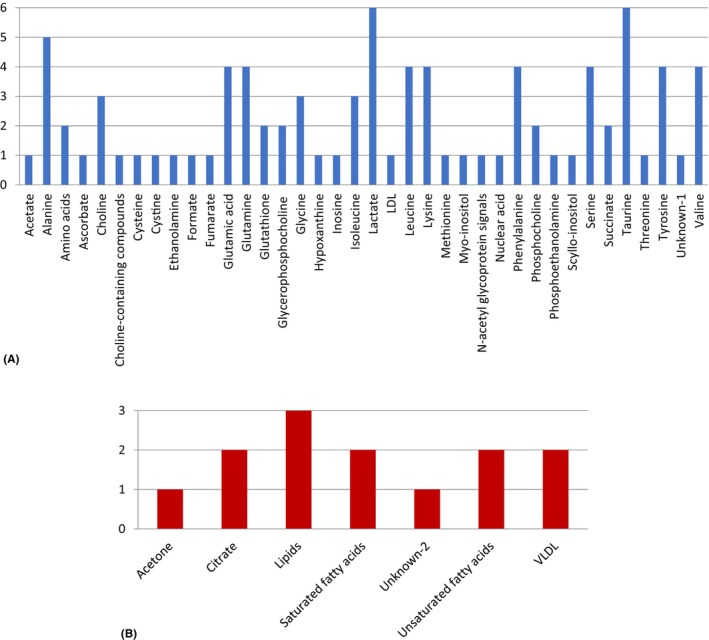
Increased metabolites (A) and decreased metabolites (B) in thyroid lesions versus normal specimens according a qualitative vote‐counting results. Each metabolite received one vote per article.

The metabolites that have been found to increase in thyroid lesions versus normal in at least three reports were: lactate, taurine, alanine, glutamic acid, glutamine, leucine, lysine, phenylalanine, serine, tyrosine, valine, choline, glycine, and isoleucine. The term “lipids” was found to be the group of metabolites that have decreased in thyroid lesions vs. normal in at least three studies. Figure 2 shows qualitative vote‐counting charts of altered metabolites in thyroid lesions versus normal specimens. The complete lists of increased/decreased metabolites for each article are provided in Tables S2 and S3.

**FIGURE 3:**
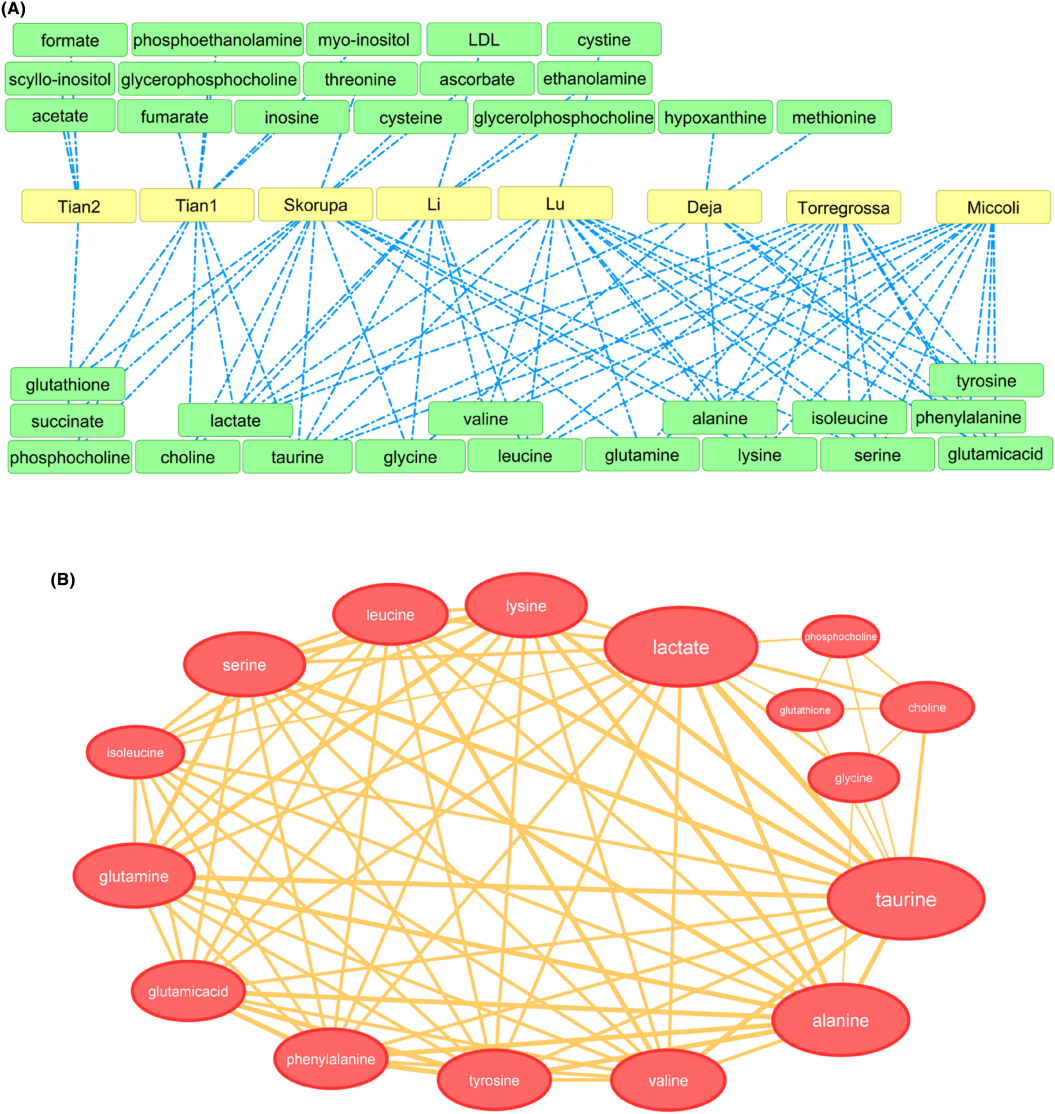
The bipartite network (A) and the bipartite network projection (B) of increased metabolites in thyroid lesions versus normal. The network was drown based on the Table S2. Compounds with a general term that were not an identifiable metabolite were removed.

According to the Table S2, a bipartite network was first constructed (Figure 3A), and then the bipartite network projection was created. It should be noted that the obtained graph was filtered according to the numbers of connections (edges) between nodes. This means that metabolites that were reported in more than one paper remained in the graph. Therefore, the graph is a weighted network, that is, the size of the nodes and the thickness of the edges indicate the number of times a particular metabolite has been reported (Figure 3B). This analysis was only carried out for Table S2.

**FIGURE 4:**
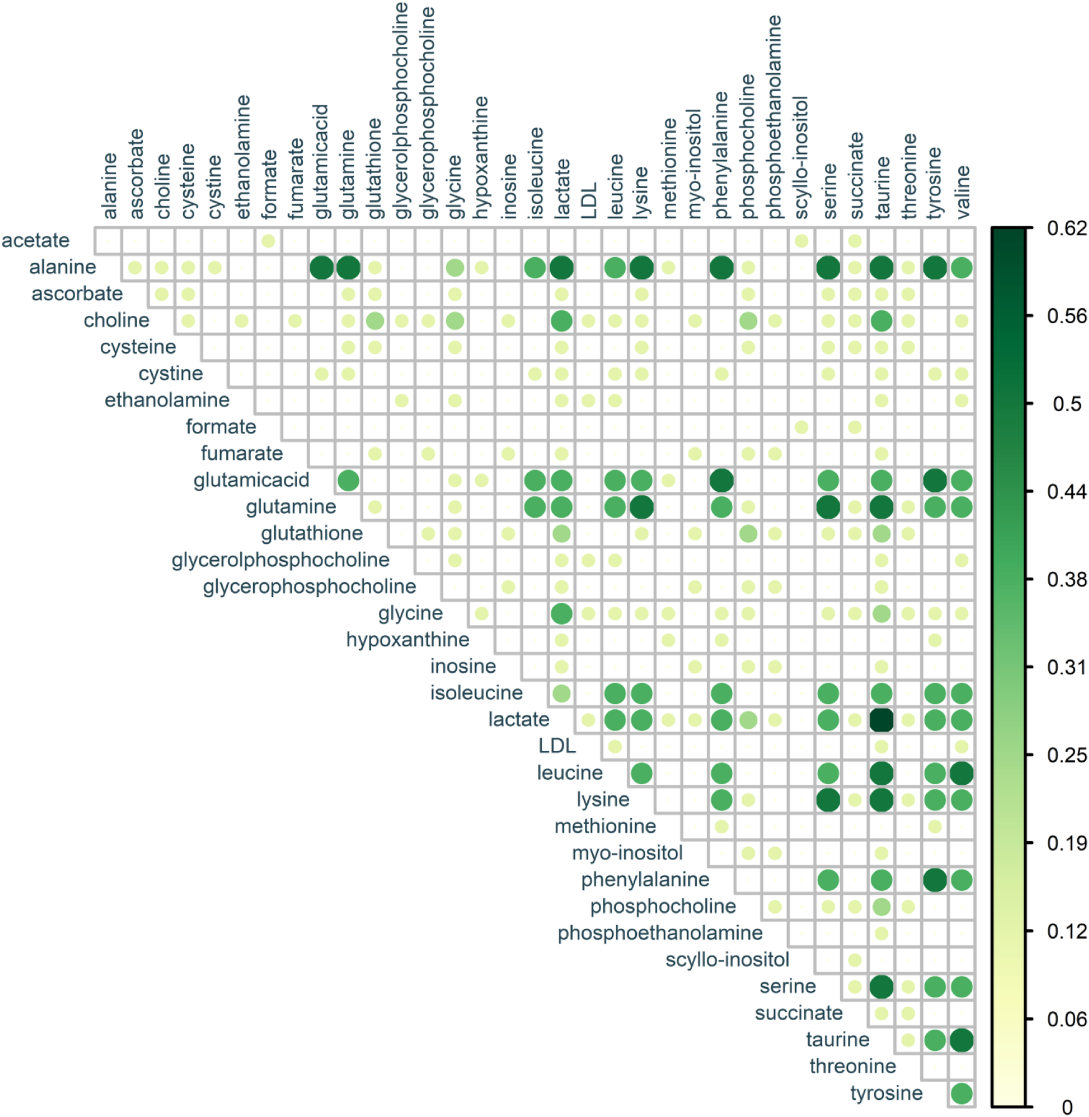
Correlation diagram of increased metabolites in thyroid lesions versus normal.

Metabolites that showed an increase in thyroid lesions were used to calculate the correlation diagram (Figure 4). This diagram shows the percentage of co‐reported frequency of the two metabolites in the articles. For example, if the correlation between two metabolites is 0.5, it means that these two metabolites were found simultaneously in 50% of the included studies. Due to the small number of articles, it was not possible to draw such a diagram for decreased metabolites.

#### 3.3.2 Altered metabolites in malignant versus benign

**FIGURE 5:**
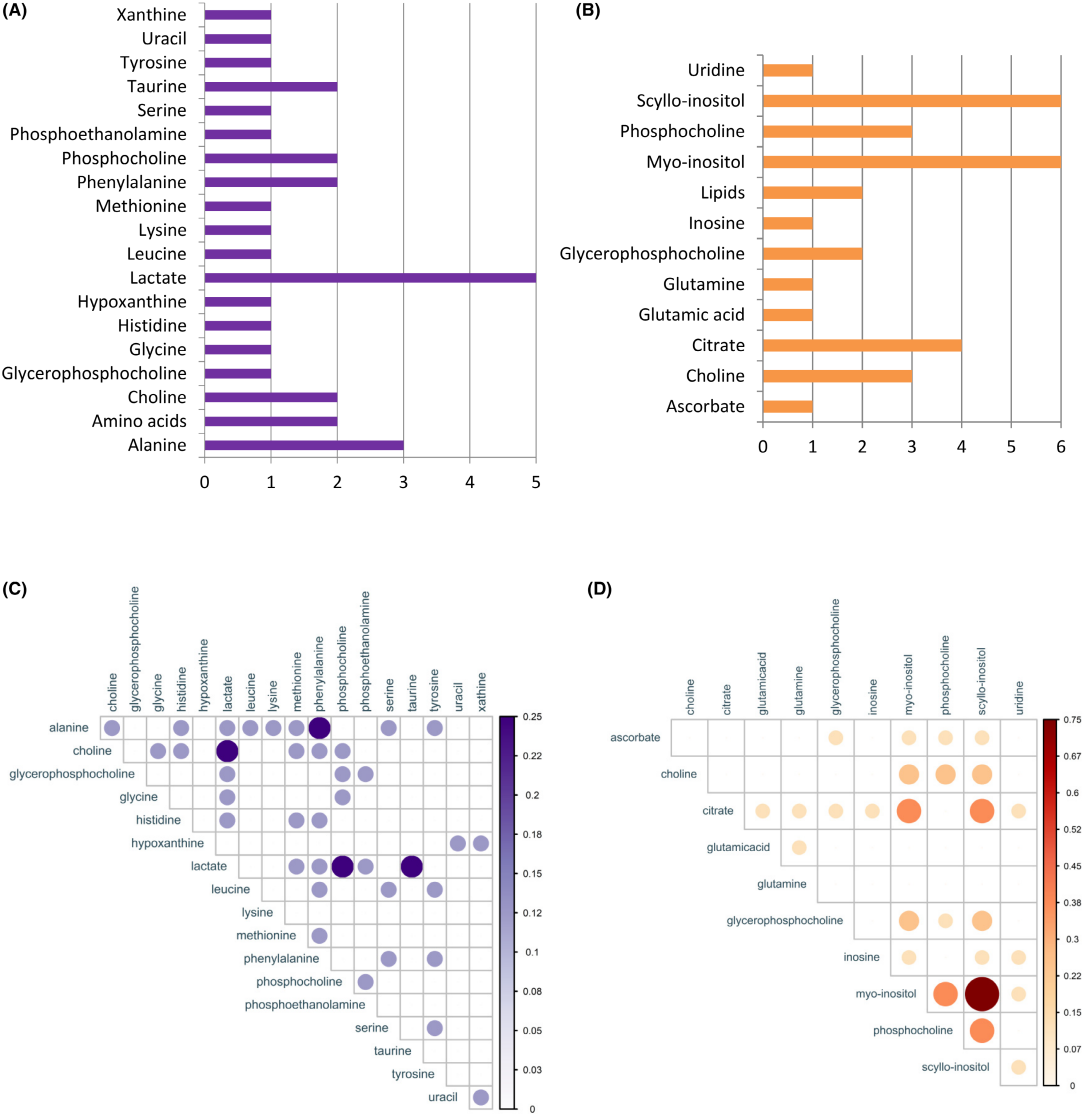
Increased metabolites (A) and decreased metabolites (B) in malignant vs. benign specimens according a qualitative vote‐counting results. Each metabolite received one vote per article. The correlation between increased (C) and decreased (D) metabolites in malignant vs. benign specimens.

In at least three reports, it has been suggested that lactate and alanine were increased in malignant versus benign thyroid tissues/FNABs. On the other hand, at least three studies have indicated that myo‐inositol, scyllo‐inositol, citrate, choline, and phosphocholine were found to be decreased in malignant thyroid tissues/FNABs in comparison to those that were benign. Figure 5A,B shows qualitative vote‐counting charts of altered metabolites in malignant versus benign thyroid specimens. The complete lists of increased/decreased metabolites for each article are provided in Tables S4 and S5. Figure 5C,D shows the correlation diagram of increased and decreased metabolites in malignancy, respectively.

### 3.4 Meta‐analysis results

Due to the constraints of the presented data in the articles, the meta‐analysis was performed only on three metabolites (lactate, alanine, and citrate) that were recognized as malignancy biomarkers in systematic review. The meta‐analysis was carried out for each metabolite on the correlation coefficients obtained from the OPLS‐DA models. Positive values indicate a relatively higher metabolite level in malignant tissues/FNABs than non‐malignant (benign or normal). Five reports related to three articles were used in the meta‐analysis: (a) the study by Tian et al.,^15^ which used intact tissue and compared malignant to benign samples (part 1 of the Tian et al. study), (b) the study by Tian et al.,^15^ which used tissue aqueous extract and compared malignant to benign samples (part 2 of the Tian et al. study), (c) the study by Li et al.,^18^ which used intact tissue and compared malignant to normal samples, (d) the study by Skorupa et al.^22^ named as part 1 of the Skorupa et al., which used intact tissue and compared malignant to non‐tumoral tissue (colloid goiter), and (e) the study by Skorupa et al.^22^ named as part 2 of the Skorupa et al., which used intact tissue and compared malignant to non‐tumoral tissue (chronic thyroiditis).

According to the meta‐analysis of lactate correlations obtained from the five OPLS‐DA models, a robust statistically significant of *p* < 0.001 for both fixed and random models with low heterogeneity among studies (*Q* = 6.75, *I* ^2^ = 40.74%, 95% CI for *I* ^2^ = 0.00–78.14, *p* = 0.1497) were obtained. The total correlation coefficient for fixed effects was 0.774 (95% CI = 0.720–0.819) and the total correlation coefficient for random effects was 0.775 (95% CI = 0.695–0.835).

Alanine was the other metabolite included in the meta‐analysis. A statistically significant findings for both fixed and random models (*p* < 0.001) with no evidence of heterogeneity (*Q* = 3.1715, *I* ^2^ = 0.00%, 95% CI for *I* ^2^ = 0.00–75.31, *p* = 0.5295) were observed. The total correlation coefficient for both fixed and random effects was 0.695 (95% CI = 0.625–0.753).

**FIGURE 6:**
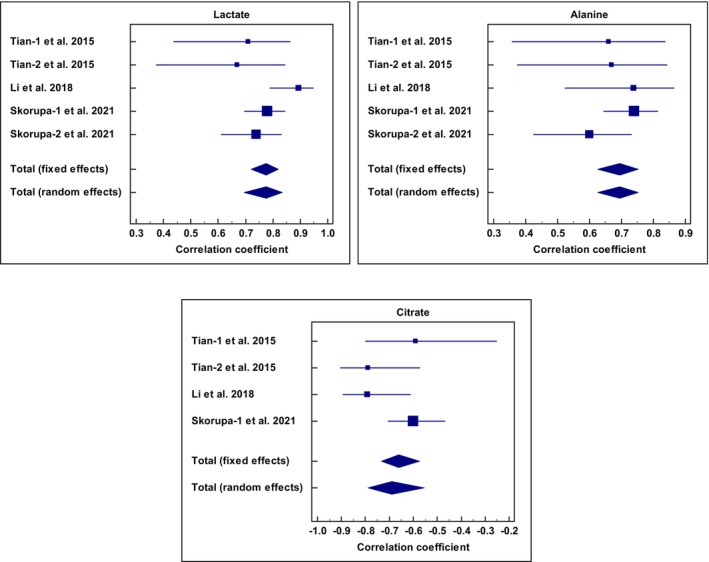
Forest plots of meta‐analysis of lactate, alanine, and citrate on the correlation coefficients obtained from the OPLS‐DA models.

The next metabolite evaluated was citrate. A statistically significant finding for both fixed and random models was *p* < 0.001. The total correlation coefficient for fixed effects was −0.661 (95% CI = −0.734 to −0.573) and the total correlation coefficient for random effects was −0.689 (95% CI = −0.789 to −0.554). The heterogeneity among studies was acceptable (*Q* = 5.4795, *I* ^2^ = 45.25%, 95% CI for *I* ^2^ = 0.00–81.76, *p* = 0.1399). Figure 6 shows the forest plots of these three metabolites.

## 4 DISCUSSION

This study conducted a systematic review and meta‐analysis on NMR‐based metabolomics studies that were performed on thyroid tissue samples or FNABs. One of our main efforts in this review was to present altered metabolites in thyroid lesions especially thyroid cancer that has been discovered so far in research those analyze metabolites using NMR spectroscopy. Introduced metabolites were presented with PubChem CID and HMDB ID (Table S1) to facilitate the comparison of the results presented here with the future reports findings.

In the systematic review, two types of comparisons were evaluated to identify altered metabolites: comparison of thyroid lesions versus normal and comparison of malignant versus benign specimens, which recognized a total of 49 compounds. Out of these 49 compounds, nine of them were not specific metabolites and were labeled as amino acids, choline‐containing compounds, lipids, N‐acetyl glycoprotein signals, nuclear acid, saturated fatty acids, unknown‐1, unknown‐2, and unsaturated fatty acids. The remaining were 40 individual metabolites classified into nine groups: (I) amino acids (alanine, cysteine, cystine, glutamic acid, glutamine, glycine, histidine, isoleucine, leucine, lysine, methionine, phenylalanine, serine, taurine, threonine, tyrosine, and valine), (II) organic acids (acetate, ascorbate, citrate, formate, fumarate, lactate, and succinate), (III) choline compounds (choline, glycerophosphocholine, and phosphocholine), (IV) lipoproteins (low‐density lipoprotein and very‐low‐density lipoprotein), (V) nucleic acid derivatives (inosine, hypoxanthine, uracil, uridine, and xanthine), (VI) carbocyclic sugars (myo‐inositol and scyllo‐inositol), (VII) amino alcohols (ethanolamine and phosphoethanolamine), (VIII) peptides (glutathione), and (IX) ketones (acetone). Out of these 40 metabolites, 13 of them have been reported only once. The metabolites that at least three studies indicated their increase in thyroid lesions were lactate, taurine, alanine, glutamic acid, glutamine, leucine, lysine, phenylalanine, serine, tyrosine, valine, choline, glycine, and isoleucine (the metabolites have been listed in order of the highest frequency). Among these, lactate and alanine were found to have increased in thyroid cancer versus benign lesions according to five and three studies, respectively. However, the only compounds that at least three articles indicated a reduction in the thyroid lesions were lipids. On the other hand, myo‐inositol and scyllo‐inositol were found to be reduced in thyroid cancer by six studies, while citrate, choline, and phosphocholine were shown to be reduced by four, three, and three articles, respectively.

Calculating the relationship between two metabolites based on the percentage of their simultaneous reporting in studies showed that the pair of lactate and taurine had the highest correlation in thyroid lesions samples. Such a correlation was observed in malignant samples between the pair of increased metabolites of alanine‐phenylalanine, choline‐lactate, lactate‐phosphocholine, and lactate‐taurine. In malignant specimens, the pair of decreased myo‐inositol and scyllo‐inositol was reported together in 75% of included studies. Other metabolites decreased in malignancy that showed a good correlation were: citrate and myo‐inositol, citrate and scyllo‐inositol, myo‐inositol and phosphocholine, and phosphocholine and scyllo‐inositol.

To begin our discussion on the metabolites that have undergone the most significant changes in thyroid lesions, particularly in cases of thyroid cancer, lactate is the appropriate starting point. One of the first and most well‐known metabolic changes in cancer cells is increased glucose consumption by tumor cells. It is now almost proven that tumor cells increase glucose uptake and produce large amounts of lactate even in the presence of oxygen.^39^ This phenomenon, known as the “Warburg effect”, explains specific aspect of cancer cell metabolism. In cancer, multiple signaling pathways affect glucose metabolism. Phosphoinositide 3‐kinase‐protein kinase B (PI3K‐AKT) signaling pathway is one of the classic pathways activated by insulin or other growth factors and stimulates glycolysis.^40^ AKT can increase glycolytic activity directly by phosphorylating hexokinase and indirectly by phosphorylating regulators of glucose transporters.^41^, ^42^ When glucose enters the oxidative phosphorylation pathway, the greatest amount of energy is produced. But during the lack of oxygen, the end product of glycolysis is lactate. However, in the presence of oxygen, cancer cells prefer the fermentation pathway of glucose to lactate. Apparently, the conversion of pyruvate to lactate by cancer cells provides the redox cofactors required for biosynthetic functions.^43^

Compared with normal or benign thyroid specimens, the increase of lactate often is paralleled by alanine. This event probably occurs through the Cahill cycle, where pyruvate is metabolized to alanine via alanine aminotransferase to be used for energy production.^44^ Genetic alterations in metabolic enzymes can cause these abnormal accumulations of intracellular metabolites. Such an event is seen in the case of the increase of the other amino acids status in tumor cells. For example, some cancer cells amplify the enzyme regulating the serine synthesis pathway (phosphoglycerate dehydrogenase) to obtain serine for de novo synthesis of purines and thymidine.^45^, ^46^ In this process, glycine is also involved alongside serine and serves as one‐carbon intermediates in the biosynthesis of nucleotides, lipids, and proteins.^47^, ^48^ Glutamine is also a very important amino acid in cancer cell metabolism. Despite being a non‐essential amino acid, glutamine plays a crucial role in the biosynthesis of multiple compounds, including glutathione, nucleotides, fatty acids, and the other non‐essential amino acids, by acting as a significant nitrogen donor.^49^

Since cancer cells are proliferating unbridled, they need more fatty acids to produce lipid membrane. In these cells, fatty acids are synthesized both through diet and from glucose, glutamine or acetate.^50^ It is possible to justify the decreased level of citrate in malignancy based on fatty acids synthesis, where citrate is transported from the mitochondria to the cytoplasm, and undergoes degradation to reduce acetyl‐CoA for the synthesis of fatty acids and oxaloacetate.^15^ The other necessary substrates for the lipid biosynthesis are choline and choline‐containing compounds. These compounds such as phosphocholine, phosphatidylcholine, and glycerophosphocholine are the main constituents of cell membranes.^51^, ^52^ However, different studies have conflicting findings regarding to status of choline in thyroid nodules. In this study, choline was found to be increases in thyroid lesions, but decreased in thyroid cancer.

Inositol is a sugar alcohol and has nine stereoisomers, among which myo‐inositol and scyllo‐inositol are the most common.^53^ These two, which play an important role in maintaining cell osmolality,^54^, ^55^ showed a significant reduction in thyroid cancer compared to benign nodules.^56^ It seems that the decrease of these two metabolites is related to the disturbance of the osmolality balance of cancer cells.

A brief meta‐analysis was performed to obtain more reliable metabolites that are altered in thyroid malignancy. Based on the meta‐analysis of correlation coefficient results, lactate and alanine were upregulated and citrate was downregulated. Accordingly, lactate, alanine, and citrate are among the biomolecules that have the potential to be used as markers for thyroid cancer. Recent research has shown that when lactate shuttles are suppressed, the ability of thyroid cancer cells to proliferation and utilize glucose is significantly reduced in a low‐glucose environment. Consequently, directing efforts towards hindering the glycolytic and lactate processing pathways could potentially be an effective and influential method of treating thyroid cancer.^57^ Another study, which focused on the diagnostic method for thyroid cancer using amino acid metabolomics in saliva, found that a combination of alanine, valine, proline, and phenylalanine can enhance the precision of early detection of thyroid cancer.^58^ The findings of the current study are in contrast to those of this particular study, which identified alanine reduction as a diagnostic condition. It is worth noting that our study examined tissue and FNABs samples, whereas the study being referenced focused on saliva samples. This distinction suggests that the metabolic changes observed in thyroid cancer may vary between local regions and the body as a whole.

The study conducted by Khatami et al. has identified citrate as the most significant diagnostic marker for thyroid cancer.^56^ The growth of different types of tumors can be inhibited by citrate, as demonstrated through multiple mechanisms. Some studies have proposed that dietary supplements which include citrate might have potential anticancer properties.^59^ All of these studies validate that there are significant alterations in metabolic pathways in thyroid lesions, particularly in cases of thyroid cancer. Nevertheless, given the dynamic nature of metabolic processes and their efficacy in response to both endogenous and exogenous factors, comprehensive investigations with a significant number of participants is necessary to determine metabolic biomarkers and apply them in clinical decisions.

## 5 CONCLUSION

Collectively, this systematic review and meta‐analysis offer a summary of the association between several metabolites and thyroid lesions/thyroid cancer based on NMR metabolomics. A bright future can be imagined for using the NMR approach in differentiating among the thyroid lesions pathology, particularly, malignancy from benignity. This study summarized the status of 49 compounds (40 metabolites) concerning thyroid lesions/thyroid cancer. In thyroid lesions, 38 metabolites were increased and seven metabolites were decreased. Ten metabolites showed an increase, while 12 metabolites indicated a decrease in thyroid cancer. We performed meta‐analysis for three metabolites can be potential biomarkers in distinguishing malignancy from benignity according to the correlation coefficients of OPLS‐DA model, detecting three significant correlation coefficients. The alteration of metabolites in thyroid lesions, particularly thyroid cancer, indicates the disruption of various metabolic pathways. Additional investigation is necessary to comprehend the fundamental metabolic pathways along with molecular mechanisms involved in thyroid cancer and convert them into clinical applications. The limited number of studies and insufficient information presented were a gap of knowledge that needs to be eliminated for future studies. If NMR‐based metabolomics is well validated for pathological differentiation of thyroid nodules, using it could potentially reduce the number of patients undergoing diagnostic surgery. Additionally, the utilization of intact tissues/FNABs without any preparations is highly advantageous for preclinical examinations and allows further evaluation of the samples after spectroscopic assessments.

## AUTHOR CONTRIBUTIONS

**S. Adeleh Razavi:** Conceptualization (lead); data curation (lead); methodology (lead); writing – original draft (lead). **Babak Khorsand:** Software (lead); visualization (lead). **Pouya Salehipour:** Investigation (supporting); methodology (supporting). **Mehdi Hedayati:** Conceptualization (supporting); investigation (supporting); methodology (supporting); validation (lead); writing – review and editing (lead).

## FUNDING INFORMATION

No financial resources were utilized in this study.

## CONFLICT OF INTEREST STATEMENT

The authors have no conflict of interest.

## Supporting information


Data S1:

## Data Availability

The data that support this study are available from the corresponding author upon reasonable request.
