# Supplementary material for: Metabolite signature of human malignant thyroid tissue: A systematic review and meta‐analysis
Source: Cancer Med. 2024 Apr 22;13(8):e7184. doi: 10.1002/cam4.7184 (PMC11033922; doi:10.1002/cam4.7184)
Supplement: Supplementary file 1 — Data S1: [file CAM4-13-e7184-s001.docx]

**Metabolite signature of human malignant thyroid tissue: A systematic review and meta-analysis**

**S. Adeleh Razavi^1^, Babak Khorsand^2,3^, Pouya Salehipour^4^, Mehdi Hedayati*^1^**

1) Cellular and Molecular Endocrine Research Center, Research Institute for Endocrine Sciences, Shahid Beheshti University of Medical Sciences, Tehran, Iran

2) Department of Neurology, University of California, Irvine, CA, USA

3) Department of Computer Engineering, Faculty of Engineering, Ferdowsi University of Mashhad, Mashhad, Iran

4) Department of Medical Genetics, School of Medicine, Tehran University of Medical Sciences, Tehran, Iran

- Instructions on how to search each of the databases:

**Embase**

The search sentences in Embase were constructed as follows:

#1 'metabolomic*':ti,ab,kw OR 'metabonomic*':ti,ab,kw OR 'metabolome*':ti,ab,kw OR 'metabolic profil*':ti,ab,kw OR 'metabolomic profil*':ti,ab,kw OR 'metabolite profil*':ti,ab,kw 85,136 results

#2 'thyroid neoplasm*':ti,ab,kw OR 'thyroid carcinoma*':ti,ab,kw OR 'thyroid cancer*':ti,ab,kw OR 'thyroid adenoma*':ti,ab,kw OR 'cancer* of thyroid':ti,ab,kw OR 'cancer* of the thyroid':ti,ab,kw OR 'thyroid nodule*':ti,ab,kw OR 'thyroid tumor*':ti,ab,kw OR 'nodular goiter*':ti,ab,kw OR 'multinodular goiter*':ti,ab,kw OR 'multi-nodular goiter*':ti,ab,kw OR mng:ti,ab,kw 85,699 results

#3 #1 AND #2 110 results

**PubMed**

The search sentences in PubMed were constructed as follows:

#1 "metabolomics"[MH] OR "metabolome"[MH] 34,236 results

#2 "metabolomic*"[TIAB] OR "metabonomic*"[TIAB] OR "metabolome*"[TIAB] OR "metabolic profil*"[TIAB] OR "metabolomic profil*"[TIAB] OR "metabolite profil*"[TIAB] 67,353 results

#3 #1 OR #2 74,878 results

#4 "thyroid neoplasms"[MH] OR "thyroid nodule"[MH] OR "goiter, nodular"[MH] 62,155 results

#5 "thyroid neoplasm*"[TIAB] OR "thyroid carcinoma*"[TIAB] OR "thyroid cancer*"[TIAB] OR "thyroid adenoma*"[TIAB] OR "thyroid nodule*"[TIAB] OR "thyroid tumor*"[TIAB] OR "nodular goiter*" OR "multinodular goiter*"[TIAB] OR "multi-nodular goiter*"[TIAB] OR "MNG"[TIAB] 62,495 results

#6 #4 OR #5 78,548 results

#7 #3 AND #6 100 results

**Scopus**

The search sentences in Scopus were constructed as follows:

#1 (TITLE-ABS-KEY ("metabolomic*") OR TITLE-ABS-KEY ("metabonomic*") OR TITLE-ABS-KEY ("metabolome*") OR TITLE-ABS-KEY ("metabolic profil*") OR TITLE-ABS-KEY ("metabolomic profil*") OR TITLE-ABS-KEY ("metabolite profil*")) 94,460 results

#2 (TITLE-ABS-KEY ("thyroid neoplasm*") OR TITLE-ABS-KEY ("thyroid carcinoma*") OR TITLE-ABS-KEY ("thyroid cancer*") OR TITLE-ABS-KEY ("thyroid adenoma*") OR TITLE-ABS-KEY ("cancer* of thyroid") OR TITLE-ABS-KEY ("cancer* of the thyroid") OR TITLE-ABS-KEY ("thyroid nodule*") OR TITLE-ABS-KEY ("thyroid tumor*") OR TITLE-ABS-KEY ("nodular goiter*") OR TITLE-ABS-KEY ("multinodular goiter*") OR TITLE-ABS-KEY ("multi-nodular goiter*") OR TITLE-ABS-KEY (mng)) 100,707 results

#3 #1 AND #2 143 results

**Supplementary Table 1**. The common names, PubChem CID and Human Metabolome Database (HMDB) ID of individual compounds identified in included studies

| **#** | **Common name** | **PubChem CID** | **HMDB ID** |
| --- | --- | --- | --- |
| 1 | Acetate | 175 | HMDB0000042 |
| 2 | Acetone | 180 | HMDB0001659 |
| 3 | Alanine | 5950 | HMDB0000161 |
| 4 | Amino acids | ___ | ___ |
| 5 | Ascorbate | 54670067 | HMDB0000044 |
| 6 | Choline | 305 | HMDB0000097 |
| 7 | Choline-containing compounds | ___ | ___ |
| 8 | Citrate | 31348 | HMDB0000094 |
| 9 | Cysteine | 5862 | HMDB0000574 |
| 10 | Cystine | 67678 | HMDB0000192 |
| 11 | Ethanolamine | 700 | HMDB0000149 |
| 12 | Formate | 283 | HMDB0000142 |
| 13 | Fumarate | 5460307 | HMDB0000134 |
| 14 | Glutamic acid | 33032 | HMDB0000148 |
| 15 | Glutamine | 5961 | HMDB0000641 |
| 16 | Glutathione | 124886 | HMDB0000125 |
| 17 | Glycerophosphocholine | 657272 | HMDB0000086 |
| 18 | Glycine | 750 | HMDB0000123 |
| 19 | Histidine | 6274 | HMDB0000177 |
| 20 | Hypoxanthine | 135398638 | HMDB0000157 |
| 21 | Inosine | 135398641 | HMDB0000195 |
| 22 | Isoleucine | 6306 | HMDB0000172 |
| 23 | Lactate | 91435 | HMDB0000190 |
| 24 | LDL | ___ | ___ |
| 25 | Leucine | 6106 | HMDB0000687 |
| 26 | Lipids | ___ | ___ |
| 27 | Lysine | 5962 | HMDB0000182 |
| 28 | Methionine | 6137 | HMDB0000696 |
| 29 | Myo-inositol | 892 | HMDB0000211 |
| 30 | N-acetyl glycoprotein signals | ___ | ___ |
| 31 | Nuclear acid | ___ | ___ |
| 32 | Phenylalanine | 6140 | HMDB0000159 |
| 33 | Phosphocholine | 1014 | HMDB0001565 |
| 34 | Phosphoethanolamine | 1015 | HMDB0000224 |
| 35 | Saturated fatty acids | ___ | ___ |
| 36 | Scyllo-inositol | 892 | HMDB0006088 |
| 37 | Serine | 5951 | HMDB0000187 |
| 38 | Succinate | 160419 | HMDB0000254 |
| 39 | Taurine | 1123 | HMDB0000251 |
| 40 | Threonine | 6288 | HMDB0000251 |
| 41 | Tyrosine | 6057 | HMDB0000158 |
| 42 | Unknown-1 | ___ | ___ |
| 43 | Unknown-2 | ___ | ___ |
| 44 | Unsaturated fatty acids | ___ | ___ |
| 45 | Uracil | 1174 | HMDB0000300 |
| 46 | Uridine | 6029 | HMDB0000296 |
| 47 | Valine | 6287 | HMDB0000883 |
| 48 | VLDL | ___ | ___ |
| 49 | Xanthine | 1188 | HMDB0000292 |

**Supplementary Table 2**. Increased metabolites in thyroid lesions vs. normal tissues, according to each included study

| **Thyroid lesions vs. Normal (Increased metabolites)** | | | | | | | |
| --- | --- | --- | --- | --- | --- | --- | --- |
| **Miccoli et al, 2012** | **Torregrossa et al, 2012** | **Deja et al, 2013** | **Tian-1 et al, 2015**  **(intact tissue)** | **Tian-2 et al, 2015**  **(tissue extract)** | **Lu et al, 2016** | **Li et al, 2018** | **Skorupa et al, 2021** |
| alanine | alanine | alanine | amino acids | acetate | alanine | choline | alanine |
| glutamic acid | glutamic acid | glutamic acid | choline | amino acid | cystine | ethanolamine | ascorbate |
| glutamine | glutamine | glycine | choline-containing compounds | formate | glutamic acid | glycerophosphocholine | choline |
| isoleucine | isoleucine | hypoxanthine | fumarate | nuclear acid | glutamine | glycine | glutamine |
| leucine | lactate | lactate | glutathione | scyllo-inositol | isoleucine | LDL | glutathione |
| lysine | leucine | methionine | glycerophosphocholine | succinate | lactate | lactate | glycine |
| phenylalanine | lysine | phenylalanine | inosine |  | leucine | leucine | lactate |
| serine | phenylalanine | tyrosine | lactate |  | lysine | N-acetyl glycoprotein signals | lysine |
| taurine | serine |  | myo-inositol |  | phenylalanine | taurine | phosphocholine |
| tyrosine | taurine |  | phosphocholine |  | serine | unknown-1 | serine/cysteine |
| valine | tyrosine |  | phosphoethanolamine |  | taurine | valine | succinate |
|  | valine |  | taurine |  | tyrosine |  | taurine |
|  |  |  |  |  | valine |  | threonine |
| **Total** | | | | | | | |
| 11 | 12 | 8 | 12 | 6 | 13 | 11 | 14 |

**Supplementary Table 3**. Decreased metabolites in thyroid lesions vs. normal tissues, according to each included study

| **Thyroid lesions vs. Normal (Decreased metabolites)** | | | | | | |
| --- | --- | --- | --- | --- | --- | --- |
| **Miccoli et al, 2012** | **Torregrossa et al, 2012** | **Deja et al, 2013** | **Tian-1 et al, 2015**  **(intact tissue)** | **Lu et al, 2016** | **Li et al, 2018** | **Skorupa et al, 2021** |
| lipids | saturated fatty acids | acetone | lipids | saturated fatty acids | citrate | citrate |
|  | unsaturated fatty acids |  |  | unsaturated fatty acids | VLDL-1 | lipids |
|  |  |  |  |  | VLDL-2 |  |
|  |  |  |  |  | unknown-2 |  |
| **Total** | | | | | | |
| 1 | 2 | 1 | 1 | 2 | 4 | 2 |

**Supplementary Table 4**. Increased metabolites in malignant vs. benign thyroid tissues, according to each included study

| **Malignant vs. Benign (Increased metabolites)** | | | | | | | |
| --- | --- | --- | --- | --- | --- | --- | --- |
| **Miccoli et al, 2012** | **Torregrossa et al, 2012** | **Deja et al, 2013** | **Tian-1 et al,**  **2015**  **(intact tissue)** | **Tian-2 et al, 2015**  **(tissue extract)** | **Ryoo et al, 2016** | **Rezig et al, 2018** | **Skorupa et al, 2021** |
| lactate | lactate | alanine | amino acids | amino acids | choline | alanine | alanine |
| taurine | taurine | choline | glycerophosphocholine | hypoxanthine | glycine | leucine | lysine |
|  |  | histidine | lactate | uracil | lactate | phenylalanine |  |
|  |  | lactate | phosphocholine | xathine | O-phosphocholine | serine |  |
|  |  | methionine | phosphoethanolamine |  |  | tyrosine |  |
|  |  | phenylalanine |  |  |  |  |  |
| **Total** | | | | | | | |
| 2 | 2 | 6 | 5 | 4 | 4 | 5 | 2 |

**Supplementary Table 5**. Decreased metabolites in malignant vs. benign thyroid tissues, according to each included study

| **Malignant vs. Benign (Decreased metabolites)** | | | | | | | |
| --- | --- | --- | --- | --- | --- | --- | --- |
| **Miccoli et al, 2012** | **Torregrossa**  **et al, 2012** | **Deja et al, 2013** | **Tian-1 et al,**  **2015**  **(intact tissue)** | **Tian-2 et al,**  **2015**  **(tissue extract)** | **Ryoo**  **et al, 2016** | **Rezig et al, 2018** | **Skorupa et al, 2021** |
| choline | choline | citrate | citrate | choline | citrate | citrate | ascorbate |
| lipids | lipids | glycerophosphocholine | inosine |  | glutamic acid | myo-inositol | myo-inositol |
| myo-inositol | myo-inositol | myo-inositol | myo-inositol |  | glutamine | scyllo-inositol | phosphocholine/glycerophosphocholine |
| phosphocholine | phosphocholine | scyllo-inositol | scyllo-inositol |  |  |  | scyllo-inositol |
| scyllo-inositol | scyllo-inositol |  | uridine |  |  |  |  |
| **Total** | | | | | | | |
| 5 | 5 | 4 | 5 | 1 | 3 | 3 | 5 |
